# MoDeMol: A Decomposition-Based Evolutionary Framework for Multi-Objective Molecular Optimization

**DOI:** 10.3390/ijms27198818

**Published:** 2026-10-02

**Authors:** Chenbin Guo, Cai Dai

**Affiliations:** School of Artificial Intelligence and Computer Science, Shaanxi Normal University, Xi’an 710119, China; 20242750@snnu.edu.cn

**Keywords:** multi-objective algorithm, molecular optimization, latent-space optimization

## Abstract

Lead compound multi-objective optimization requires balancing mutually restrictive drug-like properties, yet conventional latent generative and evolutionary optimization methods suffer from structural-function misalignment, population structural redundancy and low search efficiency under limited evaluation budgets. This work proposes a decomposition-based evolutionary framework built on a score-informed latent space (MoDeMol), with four core innovations: (1) Contrastive fine-tuned Transformer-VAE latent space supervised by predicted dual-target activity scores to improve the alignment between latent spatial proximity and predicted target-activity profiles. (2) Hybrid population initialization combining lead latent perturbation and high-quality molecular bank screening to balance local scaffold retention and global high predicted-activity region exploration. (3) Adaptive resource allocation mechanism for dynamic subproblem computation scheduling to boost optimization efficiency when the property evaluation budget is limited. (4) Dynamic Morgan fingerprint similarity penalization to suppress structural redundancy and maintain diverse molecular scaffolds in the population. Extensive benchmarks on single-target GSK3β and dual-target GSK3β/JNK3 kinase optimization tasks demonstrate that MoDeMol outperforms MolFinder, JANUS and MOMO in success rate, Pareto hypervolume and qualified molecular yield. Quantitative latent-space analyses, qualitative visualization, scaffold tracking, and ablation experiments provide complementary evidence for the contributions of the proposed components. The proposed framework addresses representation alignment, structural redundancy, and search-resource allocation within a computational multi-target lead-optimization workflow under limited evaluation budgets.

## 1. Introduction

Molecular optimization is a core stage in transforming promising lead compounds into viable drug candidates [1,2]. Computational methods have supported small-molecule drug discovery for decades, and now contribute to multiple stages of the drug design process [3]. Starting from an initial lead compound, medicinal chemists need to improve potency and selectivity while retaining key molecular interactions and structural features [4,5]. Drug-likeness, synthetic accessibility, and safety must also be taken into account throughout this process [6,7]. This requirement is particularly challenging because drug-like chemical space is vast, sparsely sampled, and unevenly explored [8,9]. Furthermore, minor structural modifications can lead to dramatic changes in biological activity, which makes reliable lead improvement more difficult [10]. Experimental synthesis and testing can only assess a tiny fraction of all plausible molecular structures, leading to long development cycles and reduced research productivity [2,11]. As a result, computer-aided drug design, machine learning, and generative modeling have become important tools for prioritizing and optimizing molecules [12]. Reviews of these technologies highlight their broad potential in molecular generation, property prediction, and decision support [13,14]. However, their practical reliability is still restricted by predictor uncertainty, limited data coverage, issues with molecular validity, synthetic accessibility challenges, and inconsistent evaluation protocols [15,16]. Therefore, effective molecular optimization must reconcile conflicting properties, maintain useful structural diversity, and explore chemical space within a limited evaluation budget [17].

Existing computational methods follow two broad paradigms. Generative approaches learn molecular distributions and sample candidate structures. Junction Tree VAE represents molecules as trees of chemical substructures, improving validity while learning a continuous latent representation for property optimization [18]. GENTRL and REINVENT convert property scores into optimization signals that bias generation towards regions satisfying activity, synthesizability, or other design criteria [19,20,21]. IDOLpro instead uses differentiable property scores to guide diffusion-based generation while balancing binding affinity and synthetic accessibility [22]. Search-based approaches start from existing molecules and iterate through structural modification, property evaluation, and candidate selection. MolFinder applies evolutionary search over SMILES and retains improved molecules while using populations to explore distinct chemical regions [23]. JANUS combines genetic operations on SELFIES with parallel-tempered populations to identify high-scoring molecules through iterative generation and fitness-based selection [24]. Generative models exploit learned structural regularities, improving validity and sampling efficiency, but remain constrained by training coverage and conditional guidance. Search methods readily incorporate non-differentiable evaluators and iteratively improve a given lead, yet depend strongly on search operators and evaluation budgets.

Molecular optimization can be implemented in either discrete chemical space or continuous latent space. Discrete approaches implement direct structural modification on SMILES strings, SELFIES, molecular graphs, and chemical fragments. Graph-based genetic algorithms generate candidate molecules via crossover and mutation operations performed on molecular graphs [25]. Graph-based Molecular Pareto Optimization similarly conducts genetic operations on molecular graphs and screens candidate molecules according to multi-property evaluation results [26]. Although these discrete operations directly correspond to structural modifications, discrete editing can induce abrupt changes in molecular properties, and requires specialized constraint rules to guarantee the chemical validity of generated molecules. Latent-space-based methods first encode molecules into continuous vector representations, and then adjust these representations via interpolation, perturbation, gradient descent, query-based updating, or population search. For example, Gomez-Bombarelli et al. optimized latent vectors using gradient information derived from differentiable property predictors [27]. QMO estimates the update direction of latent vectors based on property queries of decoded molecules [28], while MolSculptor guides the movement of points in latent space through diffusion-based transformation and surrogate models [29]. Continuous representations simplify the direct manipulation of inherently discrete molecular structures, and are compatible with well-established numerical optimization and evolutionary optimization methods. Nevertheless, the effectiveness of latent-space-based methods depends critically on how well the learned latent space organizes molecular structures and their associated physicochemical and biological properties.

Latent spaces for molecular search are typically learned primarily through molecular reconstruction tasks. As a result, the neighborhoods defined in these spaces reflect structural similarity, but do not necessarily group molecules with similar functional properties into adjacent regions. This means perturbation, crossover, and evolutionary search operations performed in these spaces lack explicit guidance toward target functional properties. This limitation is particularly prominent in target-specific molecular optimization, where highly structurally similar compounds targeting the same biological target can show significantly different activities or potencies [10]. Therefore, in a reconstruction-driven latent space, structural proximity does not equate to activity proximity. The changes in molecular activity caused by local interpolation or perturbation are hard to predict in advance, which weakens property-oriented guidance and ultimately reduces the efficiency of molecular search.

Early molecular optimization often focuses on a single molecular property. For example, Gomez-Bombarelli et al. updated latent vectors along the gradient direction output by a property predictor to optimize a predefined molecular property [27]. This formulation gives a clear search direction for single-objective optimization. However, lead optimization requires simultaneous consideration of multiple criteria, including target activity, drug-likeness, synthetic accessibility, and structural similarity to the initial lead compound. A widely adopted strategy combines multiple property scores into a single scalar objective before updating latent vectors or generative policies. For instance, QMO transforms multiple property objectives into a query-based optimization function, and estimates update directions based on aggregate evaluation of decoded molecules [28]. Similarly, REINVENT combines activity, physicochemical properties, and structural constraints into a composite scoring function to guide its generative policy [21]. While scalar aggregation is straightforward to implement, it requires predefined combination rules and relative priority weights. Moreover, the final optimization result is sensitive to the choice of weights, and this approach may also obscure the diverse trade-off relationships between different properties [4,6].

Multi-objective evolutionary algorithms use populations to explore different preference directions in parallel and advance search using separate objective evaluations. They therefore avoid collapsing all objectives into a fixed scalar score. Selection and update mechanisms vary across algorithms. Dominance-based methods typically combine non-dominated sorting with crowding measures to balance convergence and distribution. Decomposition-based methods use weight vectors to transform the problem into scalar subproblems with distinct preferences and coordinate their search [26,30]. A single run can consequently yield candidates spanning different property trade-offs. MO-LSO iteratively retrains a generative model using Pareto efficiency, progressively biasing it towards regions favored across multiple properties [31]. MolSculptor combines latent diffusion transformations with NSGA-II selection to explore trade-offs between multitarget activity and molecular constraints [29]. These methods produce non-dominated candidates across preference regions, broadening objective-space coverage and retaining diverse property compromises for downstream selection.

Practical drug optimization requires not only diverse property trade-offs but also diversity in chemical structures and molecular scaffolds. Studies distinguish Pareto diversity from molecular structural diversity. The former describes distribution in objective space, whereas the latter describes coverage across regions of chemical structure space. Improving one dimension does not necessarily improve the other [4,32]. In lead optimization, MOMO likewise evaluates candidate sets in terms of property improvement, diversity, and novelty [33]. If environmental selection considers only objective performance, identical molecules and their close analogues may be repeatedly retained, concentrating the population in a few structural classes. Environmental selection must therefore explicitly control structural redundancy.

Search-based molecular optimization is constrained by finite computational resources for candidate generation, search-space maintenance, and property evaluation. When evaluation involves complex predictors, molecular docking, or other expensive calculations, the query budget directly limits the explorable chemical space. Sample efficiency is therefore central to practical molecular optimization [17]. QMO estimates latent-space update directions from limited property queries, reducing the evaluations needed to reach an optimization target [28]. Adaptive-space-search molecular evolution constructs lead-specific fragment spaces and expands them using high-quality molecules found during optimization [34]. This design reduces inefficient exploration caused by irrelevant fragments and improves access to previously unseen chemical regions. These studies indicate that limited resources should prioritize search regions or operations with greater improvement potential. In multi-objective evolution, each new offspring generally requires property evaluation, so reproductive opportunities determine the budget assigned to each search direction. Because recent improvement potential varies across preference directions, uniform allocation can waste resources on directions with low expected returns. Reproductive opportunities should therefore adapt to recent search performance.

To address these challenges, we introduce MoDeMol, a decomposition-based multi-objective evolutionary framework operating in a score-informed latent space for lead optimization. MoDeMol fine-tunes a pretrained latent space using molecular score relationships, aligning similarity in activity scores with similarity in latent representations. Molecules with compatible properties are thus more likely to acquire related latent representations, providing more stable property guidance for linear interpolation and local mutation. MoDeMol then uses weight vectors to decompose the multi-objective problem into collaborative subproblems with distinct preferences [30]. It selects parents with similar property trade-offs from neighboring subproblems and generates offspring through latent-vector interpolation and local mutation. During environmental replacement, fingerprint similarity penalization (FSP) reduces structural redundancy. Adaptive resource allocation (ARA) dynamically adjusts reproductive opportunities across search directions. MoDeMol thereby integrates property-aware representation, multi-objective latent-space search, structural diversity, and efficient resource use within a unified framework.

## 2. Results

### 2.1. Experimental Setup

To validate the effectiveness of MoDeMol in multi-objective molecular optimization, we evaluated the method on two representative tasks. The 100 lead molecules used for benchmark testing were excluded from the datasets used for model pretraining and fine-tuning, as well as from the high-quality molecular bank used for population initialization.Each task starts from a given lead molecule and simultaneously optimizes multiple drug-related properties while preserving structural similarity to the lead, including drug-likeness (QED), normalized synthetic accessibility (SA_norm), predicted target activity, and molecular similarity. The four- and five-objective settings were designed to assess optimization performance under different objective combinations and levels of task difficulty.

#### 2.1.1. Optimization Tasks

This study defines two multi-property molecular optimization tasks to evaluate MoDeMol under different property combinations. Both tasks use a given lead molecule as input and require the generated molecules to improve multiple drug-related properties while maintaining structural similarity to the lead. Depending on the task, the objectives include drug-likeness, normalized synthetic accessibility, single- or dual-target inhibitory activity, and structural similarity to the lead molecule.

**Task 1 is a single-target inhibitor optimization task.**

This task aims to identify candidates with favorable drug-likeness, synthetic accessibility, and predicted GSK3β activity scores. It simultaneously optimizes four objectives: QED, the predicted GSK3β activity score, SA_norm, and structural similarity to the lead molecule. Target-activity scores are obtained from a prediction model, whereas QED, SA_norm, and similarity are calculated using cheminformatics tools. This task evaluates the algorithm’s ability to optimize molecular properties together with a single-target prediction score.

**Task 2 is a dual-target inhibitor optimization task.**

This task jointly optimizes predicted GSK3β and JNK3 activity scores alongside drug-likeness, synthetic accessibility, and structural similarity. The five objectives are QED, the predicted GSK3β activity score, the predicted JNK3 activity score, SA_norm, and lead similarity. Compared with Task 1, this setting introduces an additional target-activity objective and evaluates MoDeMol’s ability to manage more complex property trade-offs.

Both GSK3β and JNK3 have been implicated in neurodegenerative disease processes. Previous studies link GSK3β to amyloid and tau pathology [35], and JNK3 to neuronal stress and tau abnormalities [36]. These findings provide a biological rationale for exploring simultaneous modulation of the two targets. Here, GSK3β/JNK3 was used as a computational multi-objective optimization task aimed at simultaneously increasing predicted activity scores for both targets, rather than selectivity between them. This task did not assess selectivity against other targets or validate synergistic effects or therapeutic efficacy of dual targeting.

#### 2.1.2. Property Evaluation and Success Criteria

In this study, the predicted activity labels for GSK3β and JNK3 were obtained using the corresponding prediction models provided by the Therapeutics Data Commons (TDC) and were used during training of the score-informed latent-space model. The outputs of the TDC models are treated throughout this study as predicted activity scores rather than experimentally measured biological activities.

To further assess the final generated molecules and reduce the dependence of result interpretation on a single predictive model, we additionally used CombiMOTS [37] to re-evaluate the candidate molecules generated by MoDeMol and the baseline methods after optimization. CombiMOTS was used only for this supplementary post-optimization assessment and was not involved in model training or evolutionary search.

All methods used the same property-evaluation procedures. Predicted GSK3β and JNK3 activities were obtained from the corresponding activity models, whereas QED, SA_norm, and molecular similarity were calculated using cheminformatics tools. A generated molecule was considered successful only when it simultaneously satisfied every task-specific threshold listed in Table 1. These thresholds were adopted as unified computational screening criteria to define a common target region for all methods, rather than as absolute pharmacological cutoffs. They jointly account for drug-likeness and synthetic accessibility, predicted target activity, and structural relevance to the lead molecule while retaining room for structural exploration.

To further assess the quality of generated structures, we performed supplementary structural screening after optimization. This included RDKit sanitization, PAINS A/B/C [38] and Brenk undesirable-substructure filters, and checks for formal charges, isotopes, valence, and radicals. We also performed stereochemical cleanup and isomeric SMILES round-trip parsing checks. In this supplementary analysis, candidates were counted as post-screening successes only if they passed all structural checks and satisfied the original five-objective thresholds. This analysis assessed computational screening quality and did not establish drug relevance, synthetic feasibility, or experimental activity.

#### 2.1.3. Comparison Methods

MoDeMol is a decomposition-strategy-based multi-objective evolutionary molecular optimization method that can synchronously optimize multiple drug-related properties without relying on paired labeled optimization data. To comprehensively evaluate the effectiveness of MoDeMol in multi-objective molecular optimization tasks, this study selects three representative molecular optimization methods as comparison methods, namely JANUS [24], MolFinder [23], and MOMO [33]. The selected methods cover Pareto-optimization-based multi-objective molecular generation, genetic-algorithm-based molecular search, and latent-space-based multi-objective molecular optimization, enabling validation of MoDeMol from different perspectives, including property optimization capability, structural diversity, and search efficiency. The experimental tasks in this study include single-target inhibitor optimization and dual-target inhibitor optimization, with optimization objectives covering QED, SA_norm, GSK3β, JNK3, and structural similarity to the lead molecule. JANUS is a genetic-algorithm-based molecular optimization method. It maintains populations with different search tendencies through a parallel-tempered genetic algorithm to balance exploration of new structures and exploitation of high-scoring molecules. In the experiments of this study, JANUS retains its original scalarized optimization form, that is, multiple objectives are integrated into a comprehensive score in a preset manner, and candidate molecules are selected and updated according to this score. For Task 1, the weighted-sum coefficients for QED, SA_norm, GSK3β, and lead similarity were set to (0.20,0.20,0.40,0.20). For Task 2, the coefficients for QED, GSK3β, JNK3, SA_norm, and lead similarity were set to (0.15,0.30,0.30,0.15,0.10), selected from five candidate weight settings. Therefore, JANUS can serve as a representative evolutionary molecular optimization method based on a scalarized objective function for comparison with MoDeMol in multi-objective decomposition search and candidate-set coverage. MOMO is a multi-objective molecular optimization method based on a pretrained encoder–decoder network. It first maps molecules from discrete representations into a continuous latent space, then performs evolutionary search in the latent space, and uses a Pareto optimization strategy to select high-quality molecules in discrete chemical space. MolFinder is a genetic-algorithm-based molecular optimization method using SMILES representations. It directly performs crossover, mutation, and selection in the discrete molecular representation space to search for candidate molecules with desired properties. Because MolFinder does not rely on a continuous latent space but performs evolutionary search in SMILES space, it can serve as a representative traditional discrete-space genetic-algorithm molecular optimization method. In this study, we used the Pareto-based multi-objective version of MolFinder rather than converting multiple objectives into a fixed weighted-sum score. Therefore, no scalarization weights were used for MolFinder.

#### 2.1.4. Evaluation Metrics

To comprehensively evaluate the performance of MoDeMol in multi-objective molecular optimization tasks, this study uses four evaluation metrics to compare different methods: success rate (SR), hypervolume (HV), structural diversity (Diversity), and the total number of successful molecules (Number). SR measures whether a method can stably optimize different lead molecules into the target region satisfying preset property requirements. HV evaluates the overall quality and coverage of the generated candidate molecule set in multi-objective space. Diversity measures structural differences among successfully optimized molecules. Number counts all successful candidate molecules generated across the test set. These metrics jointly enable comprehensive evaluation of different methods from multiple aspects, including optimization success, Pareto solution-set quality, structural diversity, and generation effectiveness.

Success rate (SR) measures whether different methods can optimize input lead molecules into the target region. A lead molecule is considered successfully optimized if a method generates at least one candidate satisfying all task-specific property thresholds. Let the test set contain *N* lead molecules, of which $N_{s}$ are successfully optimized. SR is defined as

(1) $$\mathrm{SR}=\frac{N_{s}}{N}$$

where $N_{s}$ denotes the number of lead molecules for which at least one successful candidate is generated, and *N* denotes the total number of lead molecules in the test set.The success thresholds are listed in Table 1. A generated molecule is considered successful only if it satisfies all thresholds for the corresponding task.

Hypervolume (HV) measures the quality and objective-space coverage of the final valid candidate set for each lead molecule. This set includes candidates regardless of whether they satisfy all success thresholds. Let this set be *S*, with cardinality |S|. HV is calculated as

(2) $$\mathrm{HV}=\psi \left(\overset{\left|S\right|}{\underset{i=1}{⋃}}v_{i}\right)$$

where ψ denotes the Lebesgue measure, *S* denotes the successfully optimized molecule set, ∣S∣ denotes its cardinality, and $v_{i}$ denotes the objective-space region dominated by the *i*th molecule relative to the reference point. Following the recommended practice for fair HV comparison [39], the reference point was set to (0,0,0,0) for Task 1 and (0,0,0,0,0) for Task 2, with the same setting used for all compared methods. A larger HV indicates better overall objective performance and broader objective-space coverage.

Structural diversity (Diversity) measures structural differences among successfully optimized molecules. We calculate intermolecular similarity using the Tanimoto coefficient of Morgan fingerprints and use intermolecular dissimilarity as the diversity measure. For the successful molecule set S generated for a lead molecule, structural diversity is the average pairwise dissimilarity:

(3) $$\mathrm{Diversity}=\frac{2}{\left|S|(|S|-1\right)}\sum_{i<j}\left(1-\mathrm{Sim}(x_{i},x_{j})\right)$$

where $x_{i}$ and $x_{j}$ denote two distinct molecules in the successful set *S*, and $\mathrm{Sim}(x_{i},x_{j})$ denotes their Tanimoto similarity based on Morgan fingerprints.

Number measures the sum of successful candidate counts after deduplication within each lead-molecule task. A candidate is successful only if it satisfies all task-specific thresholds. Number is defined as

(4) $$\mathrm{Number}=\sum_{i=1}^{N}n_{i}$$

Here, N=100 is the number of evaluated lead molecules. For each lead *i*, $n_{i}$ counts successful candidates after deduplication by canonical SMILES within that lead’s candidate set. The same molecule is counted once within a lead’s task but separately across different leads when it satisfies the success criteria for each. Thus, Number is a sum of per-lead unique counts, not a globally deduplicated molecular count.

#### 2.1.5. Parameter Settings

For all methods, the same benchmark leads, optimization objectives, property evaluators, and evaluation criteria were used. Population size and the maximum number of generations were matched for population-based methods under each task. In addition, the compared methods used the same enlarged initialization candidate pool and the same prescreened molecular bank in our experimental implementation. These settings improve comparability of the nominal search conditions, but they do not imply identical total computational cost or identical numbers of predictor evaluations across algorithms. The remaining method-specific hyperparameters followed the default settings reported in the original papers or official code.

In this study, MoDeMol is configured according to task difficulty. For Task 1, the population size is set to 100, the maximum capacity of the elite archive is set to 100, and the maximum number of iterations is set to 100. For Task 2, the population size, elite archive capacity, and maximum number of iterations are set to 125, 125, and 125, respectively.

The genetic-operator parameters of MoDeMol are set as follows: crossover probability $p_{c}=1.0$ and mutation probability $p_{m}=0.5$. In the adaptive resource-allocation mechanism, the active subproblem set for the next stage is adjusted according to the evolutionary success rates observed during the most recent scheduling period. All experiments were conducted on a Linux server equipped with an 8-core Intel Xeon CPU, 23 GiB of system memory, and one NVIDIA GeForce RTX 3090 GPU with 24 GB memory. For MoDeMol, the wall-clock time for optimizing 100 lead molecules was 5.05 h for Task 1 and 15.86 h for Task 2, with an average runtime of approximately 11.5 min and 37.0 min per lead, respectively.

### 2.2. Validation of the Score-Informed Latent Space

We evaluated score-informed fine-tuning directly in the original high-dimensional latent space using 4000 test molecules excluded from both the pretraining and fine-tuning training sets. We performed paired comparisons of their original and fine-tuned representations. Reconstruction performance was evaluated on the same subset of 400 molecules sampled from the 4000-molecule test set for both models. The exact reconstruction rate changed from 90.25% to 90.00%, and the valid reconstruction rate changed from 99.00% to 98.75%. These small changes indicate that fine-tuning largely preserved the pretrained model’s decoding capability.

Local property consistency was quantified as the mean absolute difference in predicted activity score between each query molecule and its k nearest neighbors in latent space. For GSK3β, at k = 5, 10, and 20, this measure decreased from 0.03513, 0.03662, and 0.03856 in the original representation to 0.03206, 0.03377, and 0.03522 after fine-tuning; for JNK3, it decreased from 0.01607, 0.01707, and 0.01800 to 0.01477, 0.01516, and 0.01565, respectively. We also assessed neighborhood enrichment among high-scoring molecules. For the 130 query molecules with predicted GSK3β scores of at least 0.4, the high-score-neighbor hit rates at k = 5, 10, and 20 increased from 46.15%, 38.54%, and 31.12% to 53.69%, 46.38%, and 38.54%, respectively. For the 28 query molecules with predicted JNK3 scores of at least 0.4, the hit rates at k = 10 and 20 increased from 27.50% and 19.29% to 33.21% and 23.93%, respectively, whereas no clear improvement was observed at k = 5. These results indicate improved local consistency between the latent representation and predicted target-activity scores while reconstruction performance was largely retained.

Before evaluating the complete optimization framework, we first investigated whether contrastive fine-tuning could reorganize the latent space according to predicted GSK3β/JNK3 activity information. As a complementary qualitative analysis, we visualized the latent-space landscapes of molecules in the training dataset and compared the latent-space organization patterns under different models. Specifically, molecular latent vectors before and after fine-tuning were projected into a two-dimensional t-SNE space, and the corresponding predicted GSK3β score, predicted JNK3 score, and mean predicted dual-target score were visualized as continuous property surfaces. Because t-SNE is a nonlinear two-dimensional projection, this visualization is used to illustrate qualitative organization and is not treated as evidence of global smoothness in the original high-dimensional latent space.

**Figure 1:**
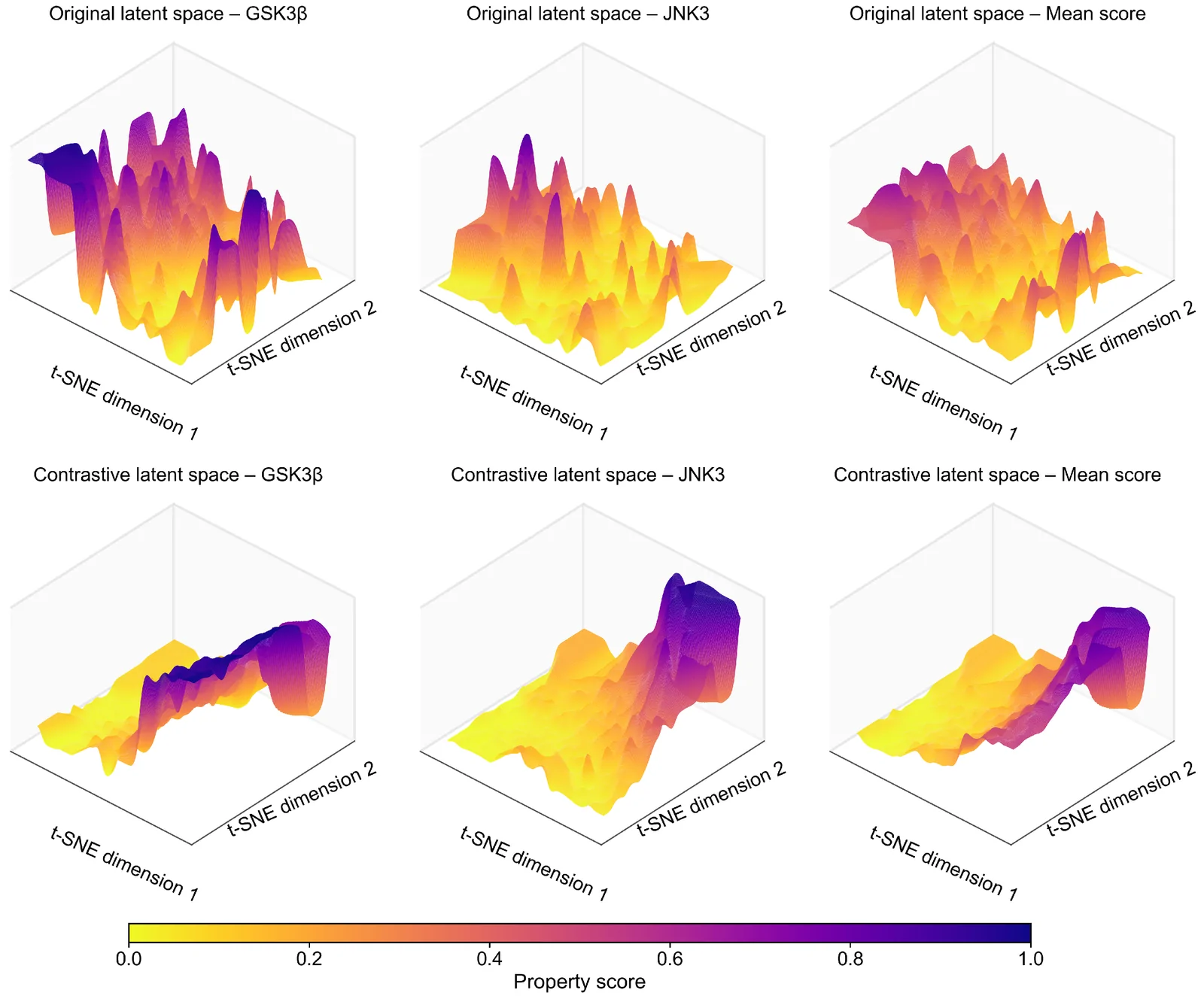
Comparison of predicted-activity landscapes in the original and score-informed contrastive latent spaces. The top and bottom rows show t-SNE projections colored by predicted GSK3β, JNK3, and mean dual-target scores before and after contrastive fine-tuning, respectively. The color scale indicates predicted activity scores from 0 to 1, and “Mean score” denotes the arithmetic mean of the GSK3β and JNK3 scores.

As shown in Figure 1, the original latent space exhibited fragmented and irregular predicted-activity distributions, with high-score regions for GSK3β, JNK3, and their mean score dispersed throughout the t-SNE projection. In contrast, the fine-tuned latent space showed a more coherent enrichment pattern in the two-dimensional projection, in which molecules with higher predicted activity occupied more spatially adjacent regions. This qualitative pattern is consistent with, but does not replace, the high-dimensional neighborhood analyses reported above.

**Figure 2:**
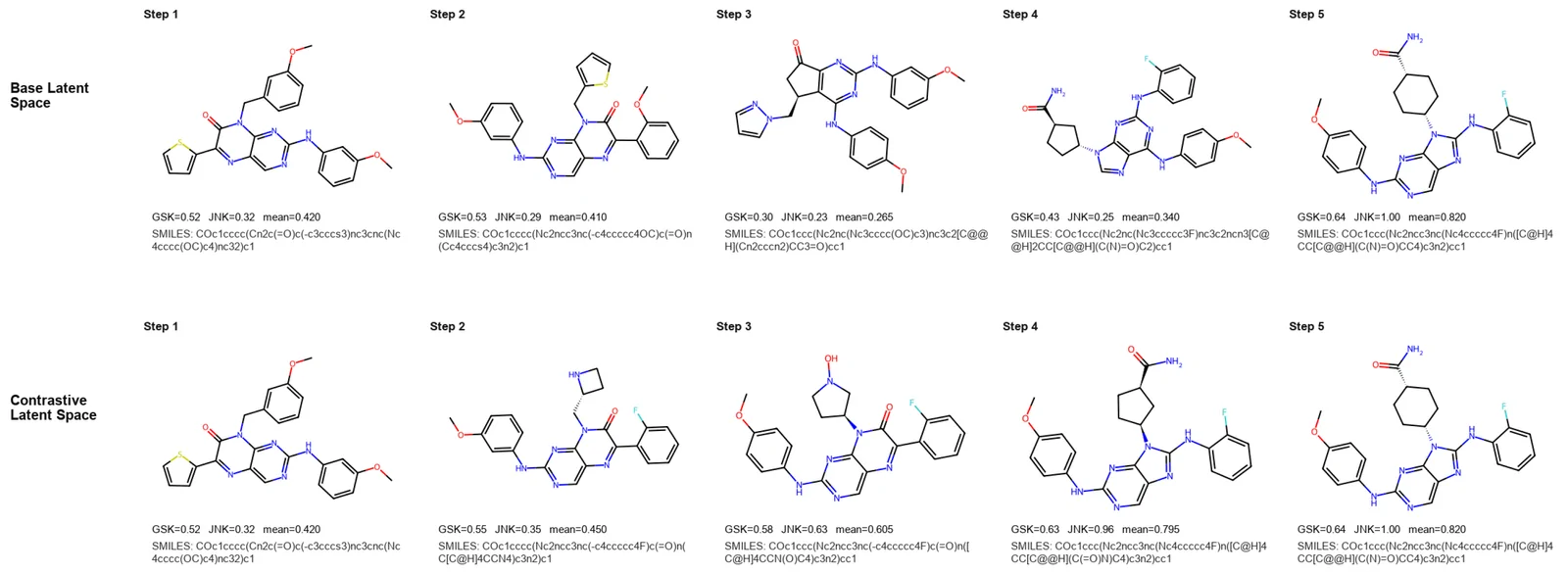
Representative molecular interpolation trajectories in the original and score-informed contrastive latent spaces. Molecular structures and predicted GSK3β, JNK3, and mean dual-target scores are shown across five interpolation steps. “GSK” and “JNK” denote predicted GSK3β and JNK3 activity scores, respectively, and “mean” denotes their arithmetic mean.

Furthermore, we examined a representative example of local latent-space interpolation rather than a global test of manifold smoothness. As shown in Figure 2, under both latent spaces, we selected a source molecule with a moderate mean predicted dual-target score and a target molecule with a high mean predicted score, and constructed a representative interpolation trajectory between them. In the original latent space, interpolated molecules exhibited unstable predicted-activity variations, with the mean score fluctuating from 0.420 to 0.410, then decreasing to 0.265 and 0.340, before finally reaching 0.820 at the target endpoint. In contrast, interpolation in the contrastively fine-tuned latent space showed a more progressive transition, with the mean predicted score increasing sequentially from 0.420 to 0.450, 0.605, 0.795, and finally 0.820 along the interpolation trajectory.

For this representative trajectory, the score-informed latent space showed a more progressive change in predicted activity than the original space; this single path is illustrative and does not establish global smoothness.

### 2.3. Comparisons on Test Tasks

Table 2 and Table 3 compare MoDeMol with MolFinder [23], JANUS [24], and MOMO [33] on two multi-objective molecular optimization tasks. The evaluation uses success rate (SR), hypervolume (HV), structural diversity (Diversity), and the total number of successful molecules (Number). Task difficulty increases from single-target GSK3β optimization to dual-target GSK3β/JNK3 optimization. This design tests whether each method can maintain success, objective-space coverage, and candidate-set scale as the number of constraints increases.

In Task 1, MoDeMol achieved the highest value for each of the four evaluation metrics, with an HV of 0.3148 ± 0.1071, an SR of 96%, a Diversity of 0.6229, and a Number of 705. MOMO ranked second, with an HV of 0.2814 ± 0.1433, an SR of 82%, a Diversity of 0.5421, and a Number of 680. MolFinder and JANUS achieved SR values of 50% and 29%, respectively, and produced 221 and 82 unique successful molecules. Thus, relative to the strongest baseline, MOMO, MoDeMol increased SR by 14 percentage points and generated 25 additional unique successful molecules.

In Task 2, all methods showed lower performance under the additional JNK3 activity constraint. MoDeMol nevertheless retained the highest HV, SR, Diversity, and Number, reaching 0.2601 ± 0.0924, 88%, 0.5797, and 755, respectively. MOMO achieved an HV of 0.1777 ± 0.1327, an SR of 56%, a Diversity of 0.4786, and a Number of 462. MolFinder and JANUS achieved SR values of 23% and 16%, respectively, and produced 139 and 71 unique successful molecules. The 32-percentage-point SR advantage and the 293-candidate yield advantage over MOMO indicate that MoDeMol maintained stronger cross-lead reliability in the more restrictive dual-target setting.

Because all methods were evaluated on the same 100 lead molecules, HV values were compared using paired statistical tests. Friedman tests showed overall differences among the four methods in Task 1 ($\chi^{2}\left(3\right)=162.588$, p<0.001, Kendall’s W=0.542) and Task 2 ($\chi^{2}\left(3\right)=205.668$, p<0.001, Kendall’s W=0.686). Post hoc two-sided Wilcoxon signed-rank tests with Holm correction showed that MoDeMol had significantly higher HV than MOMO, MolFinder, and JANUS in Task 1 (adjusted $p=1.83\times 10^{-4}$, $4.04\times 10^{-16}$, and $5.22\times 10^{-17}$, respectively) and Task 2 (adjusted $p=7.73\times 10^{-14}$, $1.47\times 10^{-17}$, and $1.32\times 10^{-17}$, respectively). The corresponding rank-biserial correlations ranged from 0.431 to 0.980 in Task 1 and from 0.861 to 0.998 in Task 2, supporting moderate-to-large and large effect sizes, respectively.

In each task, every method was evaluated on 100 lead molecules, yielding 100 paired observations for each HV comparison. Wilson 95% confidence intervals for SR were calculated for MoDeMol, MOMO, MolFinder, and JANUS, in that order. The intervals were 90.16–98.43%, 73.33–88.30%, 40.38–59.62%, and 21.01–38.54% in Task 1, and 80.19–93.00%, 46.23–65.33%, 15.84–32.15%, and 10.10–24.42% in Task 2. Confidence intervals for mean HV differences were estimated using 20,000 paired bootstrap resamples at the lead-molecule level. Relative to MOMO, MoDeMol achieved mean HV increases of 0.0334 (95% CI: 0.0187–0.0484) in Task 1 and 0.0824 (95% CI: 0.0671–0.0976) in Task 2. Diversity was summarized only across successfully optimized lead molecules, with sample sizes of 96, 82, 50, and 29 in Task 1. In Task 2, the corresponding sample sizes were 88, 56, 23, and 16, following the same method order.

### 2.4. Contributions of the Core Components

To examine whether the search mechanisms in MoDeMol contribute to final performance, we conducted ablation experiments on the dual-target five-objective task, which jointly evaluates QED, GSK3β, JNK3, SA_norm, and lead similarity. Two module-ablation variants were evaluated: MoDeMol-FSP removes molecular-fingerprint similarity penalization, whereas MoDeMol-ARA removes adaptive resource allocation. We additionally evaluated two controls: Original-latent-space uses the Transformer-VAE representation before score-informed fine-tuning, and Original-MOEA-D uses the unmodified MOEA/D search procedure. All methods were evaluated on the same 100 lead molecules.

The complete MoDeMol achieved an HV of 0.2601, an SR of 88%, a Diversity of 0.5797, and a Number of 755. Removing molecular-fingerprint similarity penalization reduced these values to an HV of 0.2387, an SR of 82%, a Diversity of 0.5216, and a Number of 554.

Removing adaptive resource allocation produced an HV of 0.2436, an SR of 74%, a Diversity of 0.6349, and a Number of 480. Thus, removing adaptive allocation increased structural diversity but reduced cross-lead success and the yield of molecules satisfying all five thresholds.

The Original-latent-space control achieved an HV of 0.2569, an SR of 88%, a Diversity of 0.6101, and a Number of 740. The Original-MOEA-D control achieved an HV of 0.2409, an SR of 80%, a Diversity of 0.5527, and a Number of 666. These controls provide reference points for the representation and search-procedure choices while keeping the lead set and evaluation criteria unchanged.

Because the complete model and the two module-ablation variants were evaluated on the same 100 lead molecules, their HV values were compared using two-sided Wilcoxon signed-rank tests, with Holm correction applied to the two comparisons. The complete MoDeMol achieved significantly higher HV than MoDeMol-FSP (Holm-adjusted $p=6.07\times 10^{-5}$; rank-biserial r=0.480) and MoDeMol-ARA (Holm-adjusted $p=5.35\times 10^{-4}$; rank-biserial r=0.399).

### 2.5. Exploration Beyond the Initial Scaffold Space

#### 2.5.1. Scaffold Statistics Across Multiple Lead Molecules

To determine whether MoDeMol explored scaffold regions beyond the initial population, we optimized 100 distinct lead molecules. For each task, we counted unique scaffolds in the final elite archive and identified those absent from the corresponding initial population. We also counted threshold-satisfying molecules that contained these new scaffolds. Scaffolds were represented as canonical, non-empty Bemis–Murcko scaffolds. A scaffold was classified as new only when it was absent from the initial population for the same lead molecule.

Across the 100 tasks, the final elite archive contained 101.13 unique scaffolds on average, of which 77.57 were new. Thus, most scaffold types retained among the non-dominated solutions were absent from the initial populations. Among the 313 molecules that satisfied all target thresholds, 255 contained a new scaffold. These results show that exploration beyond the initial scaffold set produced candidates that remained competitive under multi-objective selection and met every optimization criterion.

#### 2.5.2. Generation-Wise Tracking of Scaffold Turnover

**Figure 3:**
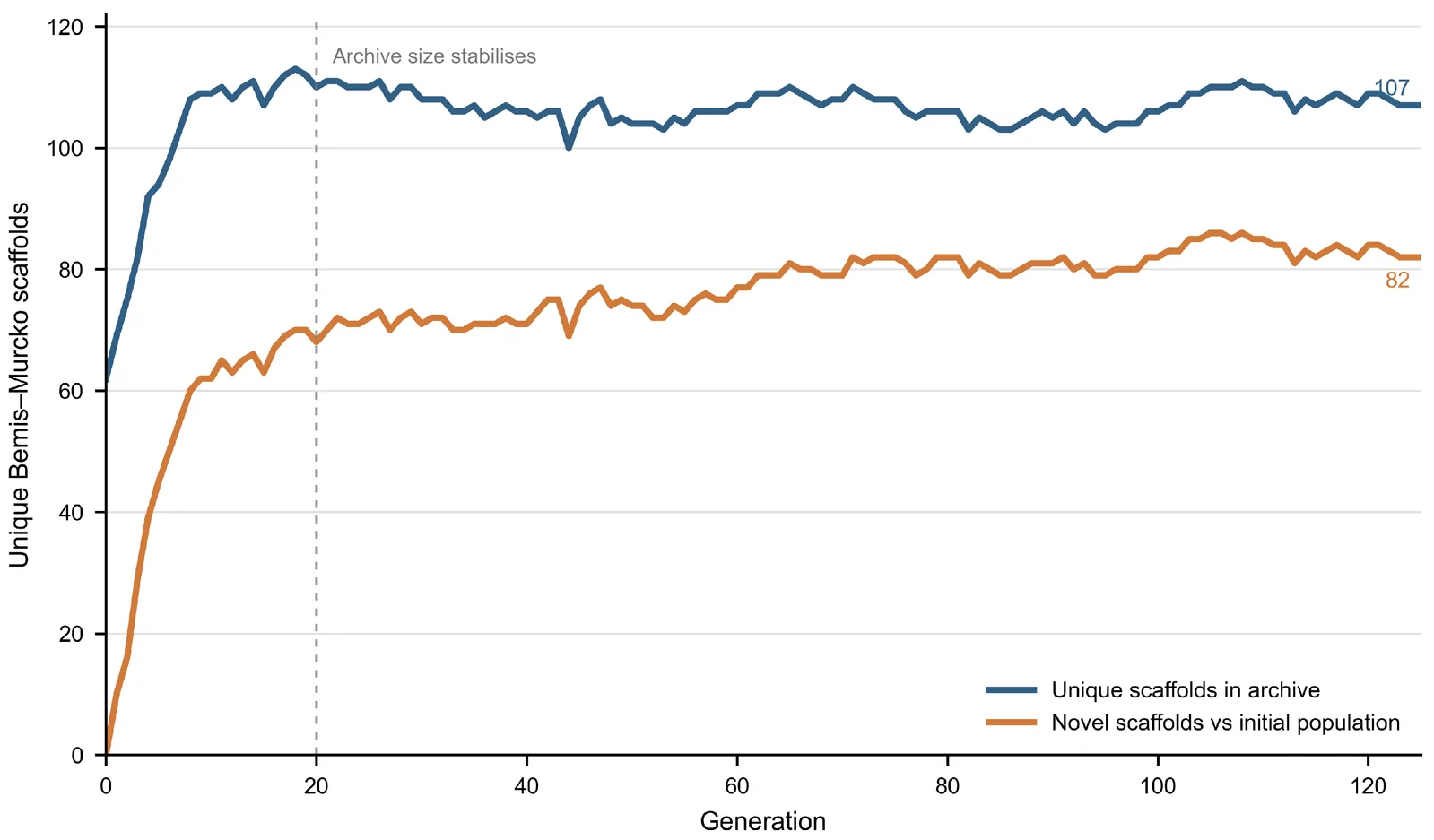
Evolution of scaffold composition in the elite archive.

To examine how archive composition evolved during optimization, we tracked all 125 generations for one successfully optimized lead molecule. Figure 3 reports two end-of-generation archive measures: the number of unique scaffolds and the subset absent from the initial population. Both measures were computed after deduplication using canonical, non-empty Bemis–Murcko scaffolds. At generation 20, the archive contained 110 unique scaffolds, including 68 new scaffolds. At generation 125, the corresponding values were 107 and 82. Although the total scaffold count remained nearly constant after generation 20 and the increase in new scaffolds slowed, the proportion of new scaffolds rose from 61.8% to 76.6%. This shift indicates continued replacement of previously retained scaffolds by structures outside the initial scaffold set.

Although both archive measures approached a plateau after generation 20, this pattern mainly reflected the archive capacity and non-dominated competition. When a new candidate entered the archive, dominated solutions or solutions removed during capacity truncation left the archive. Scaffold counts could therefore remain stable while scaffold identities continued to change. To test whether late-stage search still generated competitive structures, we additionally counted historically admitted unique scaffolds. During archive initialization and every subsequent non-dominated update, the canonical scaffold of each formally admitted candidate was recorded and deduplicated across generations. A scaffold remained in this cumulative count even if its molecule was later removed from the archive. By generation 20, 220 unique scaffolds had been admitted at least once; by generation 125, this number had reached 674. Thus, 454 previously unadmitted scaffolds entered the archive after generation 20. The plateau in archive size therefore did not mark the end of structural exploration.

The blue curve shows the number of unique scaffolds in the current archive, and the orange curve shows the subset absent from the initial population. The archive scaffold count approached a plateau after approximately 20 generations, while its scaffold composition continued to change.

#### 2.5.3. Similarity–Property Improvement–Scaffold Retention Trade-Offs

We further examined molecular similarity, property improvement, and scaffold retention using 8633 chemically screened candidate records generated by the complete MoDeMol model. These records covered 100 lead molecules; 8454 records from 98 leads had complete reference data for evaluating property improvement. Molecular similarity was measured using Tanimoto similarity between radius-2 Morgan fingerprints of each candidate and its corresponding lead. Cumulative similarity thresholds ranged from 0.2 to 0.8 in increments of 0.1. Composite property improvement was defined as the arithmetic mean of changes in QED, SA_norm, and predicted GSK3β and JNK3 scores relative to the corresponding lead. Scaffold retention was evaluated using exact Bemis–Murcko scaffold matches and Tanimoto similarity between radius-2 Morgan fingerprints of the scaffolds.

Increasing the similarity threshold from 0.2 to 0.8 reduced the retained candidate count from 3632 to 252. Mean composite property improvement decreased from 0.1311 to −0.0018, whereas exact scaffold retention increased from 10.71% to 61.51%. Descriptive molecule-level Spearman analysis showed a negative association between molecular similarity and composite property improvement (ρ=−0.5944). Molecular similarity was positively associated with scaffold similarity (ρ=0.8618) and exact scaffold retention (ρ=0.3399). Composite property improvement was also negatively associated with scaffold similarity (ρ=−0.5643). Thus, stricter post hoc similarity filtering increased scaffold retention, but the retained candidate sets generally showed smaller predicted property improvements. The threshold of 0.2 should therefore be interpreted as an exploratory minimum similarity requirement, not as evidence of retention within the original lead series.

### 2.6. Representative Molecular Evolution Case Study

**Figure 4:**
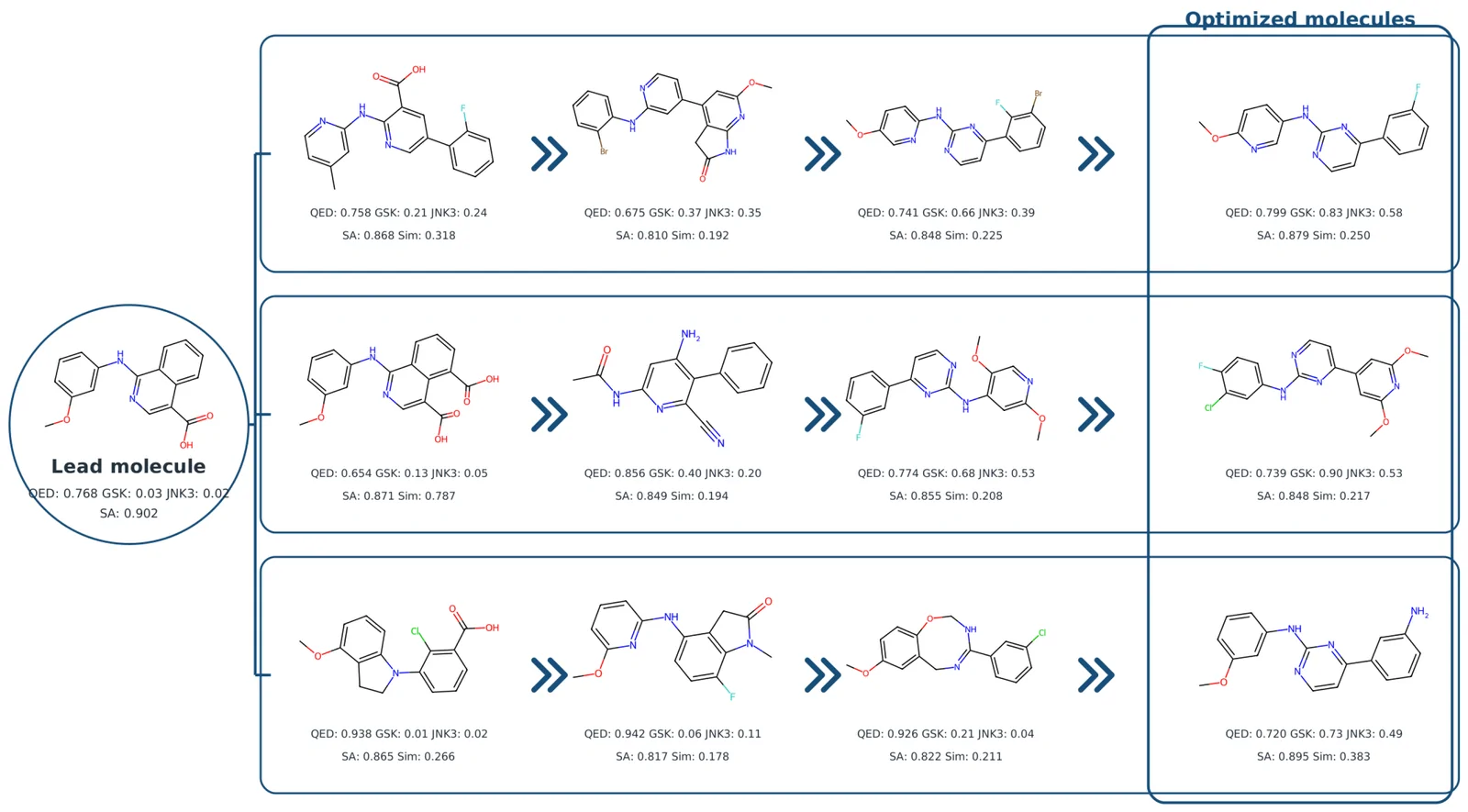
Multi-objective molecular evolution paths of MoDeMol for a representative lead molecule.

We selected a representative lead molecule to illustrate MoDeMol’s candidate-generation process during multi-objective optimization. As shown in Figure 4, the lead molecule has a QED of 0.768, predicted GSK3β and JNK3 activity scores of 0.03 and 0.02, respectively, and an SA_norm of 0.902. The molecule has favorable QED and SA_norm values but low predicted activity scores for both targets. Optimization proceeds through gradual changes to molecular scaffolds and substituents along multiple branches. Intermediate molecules show different trade-offs: some retain favorable QED or SA_norm with limited changes in predicted activity, whereas others improve one or both target scores.

The three final optimized molecules achieve GSK3β/JNK3 score pairs of 0.83/0.58, 0.90/0.53, and 0.73/0.49. Their corresponding QED values are 0.799, 0.739, and 0.720, and their SA_norm values are 0.879, 0.848, and 0.895, respectively. Compared with the lead molecule, all three candidates markedly improve both predicted target activities while retaining favorable drug-likeness and synthetic accessibility. The different activity balances among the three endpoints also demonstrate that the search produces multiple trade-off solutions rather than a single fixed optimization path.

The optimization branches produced intermediate candidates with distinct combinations of molecular properties. Some retained more lead-related structural features early in the search but showed limited improvement in the predicted GSK3β and JNK3 scores. Others reached higher predicted scores earlier, accompanied by a larger decrease in structural similarity. This representative case illustrates trade-offs among predicted target activity, drug-likeness, synthetic accessibility, and lead similarity.

## 3. Discussion

**Overall multi-objective optimization performance.** The principal finding of this study is that MoDeMol maintains a favorable balance among cross-lead success, objective-space coverage, candidate yield, and structural diversity as the task changes from a four-objective single-target setting to a more restrictive five-objective dual-target setting. The higher SR, HV, and Number values in Table 2 and Table 3 are complementary rather than redundant indicators. SR measures whether the method can obtain at least one fully qualified candidate for different lead molecules, HV reflects the quality and coverage of the successful set in objective space, and Number records the overall yield of unique candidates that cross all thresholds. MoDeMol improves these three dimensions simultaneously while retaining competitive structural diversity. Performance decreases for all methods when the JNK3 activity objective is added, confirming that the five-objective task imposes a substantially narrower feasible region. However, the smaller loss of cross-lead success observed for MoDeMol indicates that its advantage is not confined to the easier single-target setting. This distinction is important because a method can display high pairwise diversity within a very small successful set without providing reliable optimization across leads, as observed for MolFinder in Task 2. The results therefore support a bounded conclusion: under the prediction models, thresholds, and evaluation budgets used here, MoDeMol more consistently produces usable multi-property candidate sets than the evaluated alternatives, rather than merely maximizing a single aggregate score or diversity statistic.

**Score-informed latent-space organization.** The high-dimensional neighborhood analyses and the qualitative examples in Figure 1 and Figure 2 provide complementary evidence about the learned representation. A latent space trained mainly for reconstruction must preserve enough structural information to decode valid molecules, but structural proximity does not necessarily imply similarity in predicted target activity, particularly in settings where activity cliffs may occur and pose challenges for molecular machine-learning models [10,40]. Contrastive fine-tuning introduces relationships among predicted GSK3β/JNK3 scores into representation learning, encouraging molecules with compatible predicted activity profiles to occupy more closely related local neighborhoods. The quantitative reductions in nearest-neighbor score differences and the observed high-score-neighborhood enrichment support improved local alignment between the learned representation and predicted GSK3β/JNK3 activity scores. The t-SNE maps and the single interpolation trajectory are illustrative rather than evidence of global manifold smoothness. Moreover, because this study includes neither experimentally measured activities for the generated molecules nor cliff-specific evaluation metrics, these analyses do not establish mitigation or elimination of activity cliffs. The supported conclusion is limited to improved local consistency with the predicted activity scores used during fine-tuning.

**Decomposition-based search and comparison with existing approaches.** The comparative results also highlight the value of retaining multiple preference directions during lead optimization. JANUS and MolFinder conduct evolutionary search in discrete molecular representations, and their original formulations rely more strongly on predefined aggregate fitness functions [23,24]. MOMO performs multi-objective search in a learned latent space and retains non-dominated molecules [33], but its substantially lower SR and candidate yield in the dual-target task indicate that latent optimization alone is insufficient when the representation and search allocation are not explicitly adapted to the present activity constraints. By decomposing the optimization problem into cooperative scalar subproblems, MoDeMol maintains parallel search directions corresponding to different property preferences, following the general principle of decomposition-based multi-objective optimization [30]. This organization avoids forcing drug-likeness, synthesizability, target activity, and lead similarity into one fixed global weighting. Decomposition still uses weight vectors at the subproblem level, but these vectors represent a set of alternative preferences rather than one universal combination imposed on every candidate. Neighboring subproblems can exchange information, while the elite archive retains non-dominated candidates found across the different directions. It also permits different candidates to represent distinct compromises, as illustrated by the endpoints in Figure 4. Related latent or query-based methods, including QMO, MolSculptor, and MO-LSO, likewise demonstrate the usefulness of continuous representations or Pareto-aware selection [28,29,31]; the present results add evidence that property-informed representation and decomposition-based scheduling can be combined to improve cross-lead reliability under a fixed evaluation budget.

Weighted-sum scalarization has limitations in Pareto-front coverage. Although different weight vectors guide exploration of different objective trade-offs, optimizing weighted-sum subproblems alone may fail to recover some Pareto-optimal solutions on non-convex fronts. Moreover, uniformly distributed weight vectors do not guarantee uniformly distributed solutions along the Pareto front. MoDeMol’s external elite archive retains non-dominated solutions discovered during the search and uses crowding distance to maintain their distribution across objective trade-offs. However, archiving cannot guarantee discovery of regions insufficiently explored by weighted-sum search and therefore does not eliminate these coverage limitations.

**Contributions of fingerprint penalization and adaptive allocation.** The ablation patterns in Table 4 indicate that the two mechanisms address different limitations of the search. When fingerprint-similarity penalization is removed, structural diversity, SR, HV, and successful-candidate yield all decrease. Because the lead-similarity objective remains active in every variant, a population can repeatedly retain close analogues that offer small objective improvements while occupying similar structural neighborhoods. Penalizing excessive local similarity changes which candidates survive environmental replacement and helps prevent this redundancy from dominating the archive. Removing adaptive resource allocation produces a different pattern: Diversity increases, whereas SR, HV, and successful-candidate yield decrease. This pattern suggests that uniform reproduction across subproblems spends part of the evaluation budget on directions with limited recent improvement, whereas adaptive scheduling concentrates opportunities on directions that are currently producing effective replacements. The paired statistical results further support these component-level contributions, as the complete model achieves significantly higher HV than both module-ablation variants after Holm correction.

**Scaffold exploration and candidate-set value.** The scaffold and case-study analyses extend the metric-level comparison to structural behavior. Most unique scaffolds in the final archives were absent from the corresponding initial populations, and most threshold-satisfying molecules contained a new scaffold (Table 5). For the tracked lead, archive size approached a plateau early, but the proportion of new scaffolds continued to rise and the cumulative number of admitted scaffolds increased throughout the remaining generations (Figure 3). A stable archive size therefore did not imply that exploration had stopped; newly admitted candidates continued to replace dominated or truncated archive members. This distinction is relevant because Pareto diversity in objective space and chemical diversity in scaffold space are not interchangeable [4,32]. Figure 4 further shows that different evolutionary branches reach candidates with distinct balances among GSK3β/JNK3 activity, QED, SA_norm, and lead similarity. Such a set offers more decision flexibility than a single scalarized optimum: downstream evaluation can prioritize activity balance, preservation of lead-related features, or structural novelty according to experimental constraints. Nevertheless, scaffold novelty alone does not establish chemical feasibility, and these candidates should be regarded as computational hypotheses for subsequent prioritization.

**Limitations and future validation.** The conclusions of this study are limited by the use of predicted properties and fixed success thresholds. The reported GSK3β and JNK3 values depend on the corresponding activity models, so prediction error, domain shift, and uncertainty in sparsely represented chemical regions may influence both optimization and evaluation. Such uncertainty may lead to inaccurate candidate ranking and guide the search toward regions where molecular properties are overestimated by the predictive models. Applying the same evaluators to every method supports a consistent relative comparison, but some model-specific bias may be present. Although additional evaluation using predictors different from those employed during optimization-related stages helps reduce direct dependence on the original predictive models, the possibility of oracle exploitation cannot be completely excluded. During iterative optimization, the search may favor regions where predicted activity is overestimated. Therefore, the reported improvements should primarily be interpreted as computational optimization performance and require further validation in future work. The current tasks also do not directly measure target selectivity, off-target effects, chemical stability, detailed synthetic routes, or experimental assay outcomes. QED and normalized synthetic-accessibility scores are useful screening criteria, but they cannot replace expert assessment or synthesis planning [41,42]. In addition, the interpolation and molecular-evolution examples illustrate representative trajectories rather than universal smoothness of the learned latent manifold.

Structural validity checks and substructure filters can identify some overt structural problems but cannot independently establish drug relevance. Stereochemical cleanup and SMILES round-trip parsing likewise do not confirm the correctness or realizability of stereochemical configurations. Systematic retrosynthetic route validation using dedicated planning tools such as AiZynthFinder [43] and formal medicinal chemistry expert assessment have not yet been completed. Accordingly, the generated structures should be regarded as computational optimization candidates. Those passing supplementary screening satisfy the adopted computational thresholds and structural screening rules but are not validated drug candidates. More comprehensive physicochemical assessment, retrosynthetic planning, expert review, and experimental validation remain necessary.

Independent structure-based evaluation, counter-target prediction, ADMET analysis, predictive uncertainty quantification, and experimental measurements have not been systematically performed in this study. The present results therefore support multi-objective optimization performance under the specified prediction models and evaluation conditions, without establishing target selectivity, safety, or therapeutic efficacy. These assessments and experimental validation will be priorities for future work.

Future work will further validate promising candidates using molecular docking, additional independent activity predictors, and ultimately experimental assays to assess their actual biological activity. Prospective synthesis and biological assays will ultimately be required to determine whether the computational improvements translate into experimentally active and practically accessible molecules. Evaluating MoDeMol under these more expensive measurements would also provide a stricter test of whether adaptive resource allocation retains its advantage when each candidate evaluation carries a substantially higher cost.

## 4. Materials and Methods

### 4.1. Multi-Objective Lead Optimization Problem

In multi-objective molecular optimization, a candidate molecule is usually required to satisfy multiple drug-related requirements simultaneously. In this study, the property evaluation of a candidate molecule x is represented as a multi-objective function vector:

(5) $$F\left(x\right)\;=\;(f_{1}\left(x\right),\;f_{2}\left(x\right),\;\ldots ,\;f_{m}\left(x\right))$$

Here, *m* denotes the number of optimization objectives, and $f_{k}\left(x\right)$ represents the value of the *k*th objective for candidate molecule *x*. Depending on the task, the objectives include drug-likeness, synthetic accessibility, predicted target-activity scores, and structural similarity to the lead molecule.

Each candidate molecule is first checked for chemical validity using RDKit. QED and predicted GSK3β/JNK3 activity scores are then obtained using the corresponding property evaluators. Synthetic accessibility is evaluated using RDKit and subsequently normalized, while lead similarity is calculated as the Tanimoto similarity between Morgan fingerprints.

For unified optimization, MoDeMol transforms all objectives into maximization-oriented objectives. After this transformation, a larger objective-function value indicates better performance of the candidate molecule with respect to the corresponding property. The goal of multi-objective optimization is to obtain a set of candidate molecules rather than a single optimal molecule. This candidate set should achieve reasonable trade-offs among multiple properties and cover different Pareto-dominant regions as broadly as possible. Therefore, MoDeMol does not simply combine multiple objectives into a fixed weighted score. Instead, it adopts a decomposition-based optimization strategy to advance the search simultaneously along multiple objective-preference directions.

### 4.2. Overview of the MoDeMol Framework

This study proposes MoDeMol, a decomposition-based evolutionary algorithm designed for multi-objective molecular optimization. Given a lead molecule, MoDeMol first projects it into a continuous latent space and initializes a hybrid population composed of two complementary components. The first component is generated by perturbing the latent representation of the lead molecule followed by decoding, thereby preserving structural relevance to the original lead scaffold. The second component consists of high-quality candidate molecules selected from public molecular databases according to GSK3β/JNK3 activity scores, which biases the evolutionary search toward regions of the chemical space with potentially improved bioactivity profiles. The multi-objective optimization problem is then decomposed into a set of collaborative scalar subproblems by means of predefined weight vectors, such that each subproblem characterizes a distinct objective-preference direction. Throughout the iterative optimization process, each candidate molecule is evaluated with respect to multiple drug-related properties, including drug-likeness, synthetic accessibility, predicted target-activity scores, and structural similarity to the lead molecule.

The overall optimization procedure of MoDeMol comprises four principal stages. First, an initial population is constructed by combining lead-centered latent perturbations with high-scoring database-derived molecules, followed by chemical validity checking and property evaluation of the candidate molecules. Second, decomposition-based subproblems are formulated according to the weight vectors, and a scalarization function is employed to quantify the fitness of candidate molecules under different objective-preference directions. Third, active subproblems are selected at each iteration according to a dynamic resource allocation mechanism, after which parent selection, latent-space crossover, and Gaussian mutation are performed within the corresponding neighborhoods to generate offspring molecules. Fourth, during environmental selection, a neighborhood-based molecular-fingerprint similarity penalty is incorporated into the replacement criterion, enabling the algorithm to jointly account for objective performance and structural redundancy. Meanwhile, valid non-dominated candidate molecules are maintained in an external elite archive.

Within this framework, the external elite archive serves to preserve high-quality non-dominated molecules discovered during the search process, which ultimately constitute the optimized candidate set. The search efficiency of MoDeMol is further regulated by the dynamic resource allocation mechanism, which periodically reallocates evolutionary opportunities across different search directions according to the recent frequency of effective replacements produced by each subproblem. By integrating decomposition-based search, adaptive resource scheduling, fingerprint-similarity penalization, and external elite preservation, MoDeMol is able to maintain multi-objective search capability while improving structural diversity, retaining non-dominated solutions, and enhancing resource-utilization efficiency under a limited evaluation budget. The overall workflow of MoDeMol is shown in Algorithm 1.


**Algorithm 1. MoDeMol**
**Input: lead molecule **$x_{0}$**, high-quality molecular bank, objective-function set** 
*F*
**, weight-vector set** 
*W*
**, and population size** 
*N*
**,** maximum iterations *T*, neighborhood size *B*, number of active subproblems $N_{active}$, scheduling period $T_{r}$, and similarity-penalty parameters. **Output:** optimized molecular set *P* and elite archive *A*. Construct hybrid initial population $P_{0}$ by combining latent perturbations around the encoded lead molecule $x_{0}$ with latent representations of high-scoring molecular-bank candidates, and construct weight-vector neighborhoods; Evaluate *F*($P_{0}$), and initialize ideal point $z^{*}$ and nondominated elite archive *A*; Set the success counter $c_{i}$ = 0 for each subproblem and initialize uniform allocation probabilities $p_{i}$ = 1/*N*; for *t* = 1 to *T* do Generate an active-subproblem set $I_{active}$ of length $N_{active}$ by roulette-wheel sampling with replacement according to $p_{i}$; for each subproblem *i* in $I_{active}$ do Select parents from the weight-vector neighborhood; Perform latent crossover and Gaussian mutation, and decode candidate $x^{\prime }$; Discard $x^{\prime }$ and continue if it is chemically invalid; Evaluate *F*($x^{\prime }$) and update ideal point $z^{*}$; Compute scalarized fitness for the target subproblems and their neighborhoods; Compute the structural-redundancy penalty from molecular fingerprint similarity; Update subproblem representatives using the penalized update criterion; If the candidate produces a successful replacement, set $c_{i}$ = $c_{i}$ + 1; Update elite archive *A* according to Pareto dominance; end for if *t* mod $T_{r}$ = 0 and *t* < *T* then Update allocation probabilities $p_{i}$ using the success counts accumulated during the current period; Reset all success counters $c_{i}$ to zero; end if end for **return** *P*, *A*

### 4.3. Construction of Molecular Latent Space

Generative models trained primarily with molecular reconstruction objectives can establish a continuous and decodable latent space. However, the organization of such a space mainly reflects molecular structural characteristics, and proximity between latent variables does not necessarily correspond to functional similarity in a specific pharmacological task. In the context of dual-target optimization for GSK3β and JNK3, molecules with similar activity profiles may be scattered across different regions of the latent space, thereby limiting the effectiveness of local latent-space operations in generating task-relevant candidates. To address this issue, we introduce supervision from GSK3β and JNK3 activity scores into a pretrained Transformer-VAE to construct a Score-Informed Latent Space, in which the local geometry jointly captures molecular structural information and dual-target activity relationships. Meanwhile, by constraining the extent to which task-specific fine-tuning alters the original latent space, the continuity of the molecular representations and the decoding capability of the model are preserved. Consequently, neighboring latent vectors are more likely to correspond to decodable molecules with similar activity profiles, thereby providing a more task-relevant continuous search space for subsequent multi-objective evolutionary optimization in the latent space.

#### 4.3.1. Molecular Data Preparation

Molecular data were sampled from PubChem and ChEMBL. Each record contained a randomized SMILES, a canonical SMILES, and two molecular-property labels: QED and normalized synthetic accessibility (SA_norm). QED was calculated from molecular structure using RDKit and ranged from 0 to 1, with higher values indicating greater drug-likeness. SA_norm was calculated using the synthetic-accessibility score proposed by Ertl and Schuffenhauer [44] and then normalized, with higher values indicating better synthetic accessibility. After SMILES vocabulary parsing, 4,954,456 valid molecular records were retained for model training.

After constructing the general molecular latent space, the pretrained Transformer-VAE was further fine-tuned for GSK3β/JNK3 dual-target molecular optimization. The fine-tuning dataset comprised candidate molecules with GSK3β or JNK3 activity information. It contained 311,770 valid molecular records corresponding to 130,015 unique canonical SMILES.

#### 4.3.2. General Latent-Space Pretraining with Transformer-VAE

We constructed a variational autoencoder with Transformer-based encoder and decoder backbones. For each training molecule, randomized and canonical SMILES were generated as the model input and supervision target, respectively. The encoder received the randomized SMILES, whereas the corresponding canonical SMILES served as the reconstruction target. After tokenization, embedding, and positional encoding, the randomized SMILES were passed to the Transformer encoder, which produced a variable-length memory representation. Mean pooling, max pooling, and the start-token representation were concatenated to compress this memory into a fixed-length molecular representation *h*. This representation was used to estimate the mean and variance of the posterior latent distribution. A latent variable *z* was then obtained through reparameterization. The posterior latent distribution was defined as

(6) q(z∣x)=N(μ,diag(v))

where μ denotes the mean of the latent distribution and *v* denotes its variance. The latent variable was obtained using the reparameterization procedure:

(7) $$z=\mu +\varepsilon ⊙\sqrt{v}$$

Conditioned on latent variable z, the Transformer decoder autoregressively generated the canonical SMILES. To improve the representation of basic physicochemical properties, a property-prediction head was attached to the latent mean vector μ. The head predicted QED and normalized synthetic accessibility (SA_norm), enabling the latent space to encode both molecular structure and property-related information.

The model learned to recover molecular sequences through the reconstruction loss, while KL regularization constrained the latent distribution. After training, latent variables could be sampled from a standard normal distribution and decoded into new canonical SMILES. The overall pretraining objective combined the reconstruction loss, KL-divergence loss, and property-prediction loss for QED and SA_norm:

(8) $$L=L_{rec}+\lambda_{kl}L_{kl}+\lambda_{prop}L_{prop}$$

where $L_{rec}$ denotes the SMILES reconstruction loss, $L_{kl}$ denotes the KL-divergence loss, and $L_{prop}$ denotes the property-prediction loss. $\lambda_{kl}$ and $\lambda_{prop}$ are the weighting coefficients for the corresponding loss terms.

#### 4.3.3. Score-Informed Latent Space Fine-Tuning

For molecule *i*, the dual-target activity label $s_{i}={\left(s_{i}^{GSK3B},s_{i}^{JNK3}\right)}^{T}$ is a two-dimensional vector representing its predicted inhibitory activities against GSK3β and JNK3. Specifically, the canonical SMILES was scored using the GSK3β and JNK3 oracles provided by Therapeutics Data Commons (TDC) [45]. These oracles are ECFP6 random-forest classifiers trained on ExCAPE-DB activity data and used by Li et al. and Jin et al. in multi-objective molecular-generation studies. Each model returns an activity probability between 0 and 1, with higher values indicating a greater predicted likelihood of activity against the corresponding target. These scores are model-predicted activity probabilities rather than experimentally measured biological activities. The same TDC predictors were used to generate the activity-related labels for latent-space fine-tuning and to evaluate the target-activity objectives during optimization.

Fine-tuning was initialized from the pretrained model. The encoder was updated, whereas the decoder parameters remained frozen. The frozen decoder still participated in the forward pass, allowing reconstruction-loss gradients to propagate through it to the encoder and latent-mapping module without updating decoder parameters. The encoder was therefore constrained to produce latent variables that remained correctly interpretable by the original decoder. The latent-distribution mean $\mu_{i}$ was retained as the deterministic latent representation of molecule *i*. This design preserved the SMILES reconstruction capability and latent-space continuity learned during pretraining.

Within each training batch of *B* molecules, a normalized weight was calculated from the distance between property vectors $s_{i}$ and $s_{j}$:

(9) $$w_{ij}=\frac{\exp(-{∥s_{i}-s_{j}∥}_{2}^{2}/\tau_{s})}{\sum_{k=1}^{B}\exp\left(-{∥s_{i}-s_{k}∥}_{2}^{2}/\tau_{s}\right)}$$

where $w_{ij}$ denotes the property-similarity weight of molecule *i* relative to anchor molecule *j*, and $\tau_{s}$ is the property-space temperature. As the GSK3β/JNK3 activities of two molecules become more similar, $w_{ij}$ increases. These weights were then applied to latent-space similarities within the batch, yielding the continuous contrastive loss:

(10) $$L_{con}=-\frac{1}{B}\sum_{i=1}^{B}\sum_{j=1}^{B}w_{ij}log\frac{\exp({{\bar{\mu }}_{i}}^{T}{\bar{\mu }}_{j}/\tau_{z})}{\sum_{k=1}^{B}\exp\left({{\bar{\mu }}_{i}}^{T}{\bar{\mu }}_{k}/\tau_{z}\right)}$$

where ${\bar{\mu }}_{i}$ and ${\bar{\mu }}_{j}$ are the L2-normalized forms of latent mean vectors $\mu_{i}$ and $\mu_{j}$, respectively, and $\tau_{z}$ is the latent-space temperature. This loss encourages molecules with similar dual-target activity profiles to acquire similar latent representations while retaining continuous activity supervision. To prevent excessive deviation from the pretrained space, we further introduced a latent-space preservation regularizer:

(11) $$L_{reg}=\frac{1}{B}\sum_{i=1}^{B}{∥\mu_{i}^{ft}-\mu_{i}^{pre}∥}_{2}^{2}$$

where $\mu_{i}^{ft}$ denotes the latent mean produced by the fine-tuned model and $\mu_{i}^{pre}$ denotes the latent mean produced for the same molecule by the frozen pretrained model. This regularizer limits representation shifts during fine-tuning and reduces disruption of the original continuous latent-space structure by task-specific supervision.

Combining these constraints, the overall objective for latent-space fine-tuning was

(12) $$L_{fine}=\lambda_{rec}L_{rec}+\lambda_{kl}L_{kl}+\lambda_{con}L_{con}+\lambda_{reg}L_{reg}$$

where $L_{rec}$ and $L_{kl}$ are the previously defined SMILES reconstruction and KL-divergence losses, which ensure that the fine-tuned representations remain reconstructable by the frozen decoder. $L_{con}$ denotes the continuous contrastive loss, and $L_{reg}$ denotes the latent-space preservation regularizer. $\lambda_{rec}$, $\lambda_{kl}$, $\lambda_{con}$, and $\lambda_{reg}$ are the respective weighting coefficients. Joint optimization preserves structural continuity and decodability while incorporating predicted GSK3β/JNK3 dual-target activity information. The resulting score-informed latent space organizes molecules with similar predicted GSK3β/JNK3 activity profiles into more continuous local neighborhoods, strengthening the correspondence between latent proximity and predicted target-activity profiles.

### 4.4. Decomposition-Based Multi-Objective Molecular Evolution

#### 4.4.1. Population Initialization and Problem Decomposition

MoDeMol first reformulates the molecular optimization problem as an evolutionary search problem in a continuous latent space. Given a lead molecule $\;x_{0}$, the algorithm employs a pretrained molecular encoder E(·) to map its SMILES representation into a continuous latent vector:

(13) $$z_{0}\;=\;E\left(x_{0}\right)$$

where $z_{0}$ denotes the continuous representation of the lead molecule in the latent space. Unlike directly performing crossover and mutation on discrete SMILES strings, latent-space representation provides a smoother search domain, allowing molecular structural variations to be realized through continuous vector perturbations.

After obtaining the latent vector of the lead molecule, MoDeMol constructs a hybrid initial population. One subset of the population is generated by introducing random perturbations around the latent vector of the lead molecule, while the other subset is initialized from high-scoring molecules selected from public molecular databases based on their GSK3β and JNK3 scores. For the decoded candidate molecules, the algorithm first performs chemical validity checking and removes molecular structures that cannot be parsed or fail to satisfy basic chemical constraints. Molecules generated from lead-centered perturbations and high-scoring database-derived molecules are jointly evaluated according to the objective-property values and then used as the initial population for decomposition-based evolutionary search. This hybrid initialization preserves local structural relevance to the lead molecule while introducing functionally promising candidates with high GSK3β/JNK3 scores, thereby improving both initial population quality and search diversity.

To handle the conflicts among multiple objective properties, MoDeMol employs a set of weight vectors to decompose the original multi-objective optimization problem into multiple scalar subproblems. Let the set of weight vectors be

(14) $$W=\{w_{1},w_{2},\ldots ,w_{N}\}$$

where $w_{i}=\left(w_{i1},w_{i2},\ldots ,w_{im}\right)$ denotes the objective-preference vector of subproblem *i* and satisfies

(15) $$w_{ik}\geq 0,\;\sum_{k=1}^{m}w_{ik}=1$$

The weight vectors are constructed on the *M*-dimensional objective simplex to cover different trade-off directions as uniformly as possible. Accordingly, vectors satisfying the non-negativity and unit-sum constraints are generated. To enable information sharing among neighboring objective-preference directions, MoDeMol constructs a neighborhood for each subproblem according to directional similarity between weight vectors. For subproblem *i*, neighborhood B(i) contains the subproblems associated with the weight vectors closest to $w_{i}$:

(16) $$B\left(i\right)=\left\{i_{1},i_{2},\ldots ,i_{T}\right\}$$

where *T* denotes the neighborhood size. Parent selection, offspring competition, and population updating are mainly performed within this neighborhood. Through decomposition-based neighborhood collaboration, MoDeMol maintains parallel searches along multiple objective-preference directions while allowing similar subproblems to share useful molecular structural information, thereby improving coverage of the multi-objective candidate set.

#### 4.4.2. Adaptive Subproblem Selection and Offspring Generation

In decomposition-based multi-objective optimization, each weight vector defines a scalar subproblem with a distinct objective preference. Subproblems differ in their recent improvement potential, so uniformly allocating reproductive opportunities may reduce efficiency under a limited iteration budget. We therefore use an adaptive resource-allocation mechanism that tracks successful offspring replacements for each subproblem. This feedback periodically determines which subproblems receive reproductive opportunities, directing more search effort toward those with greater recent replacement success.

**Active subproblem sampling and resource scheduling**

According to the resource-allocation probabilities $p_{i}$, the algorithm uses roulette-wheel sampling to construct the active subproblem set for the current stage:

(17) $$I_{active}=\mathrm{Sample}\left(\left\{1,\ldots ,N\right\},n_{active},p\right)$$

where $\;n_{active}$ denotes the number of subproblems assigned reproduction resources at each scheduling stage. The algorithm performs parent selection, crossover, mutation, and competitive updating only for the subproblems in $I_{active}$. After each scheduling step, all success counters are reset and a new statistical period begins. This mechanism dynamically adjusts search-resource allocation according to recent evolutionary feedback instead of assigning reproduction opportunities uniformly. Subproblems that are more likely to produce effective improvements receive more search opportunities, whereas directions with fewer recent improvements receive fewer opportunities. Thus, MoDeMol improves offspring generation and population updating under a limited iteration budget.

After selecting the active subproblems, MoDeMol performs parent selection and offspring generation within their neighborhoods, allowing neighboring objective preferences to share candidate information. Offspring latent vectors are decoded into molecules, checked for chemical validity, and evaluated against the objectives. Valid offspring are then considered for population updates and inclusion in the external elite archive.

For each active subproblem i, MoDeMol first selects parent molecules from its neighborhood B(i). This neighborhood comprises subproblems whose weight vectors are close to the current weight vector $w_{i}$; therefore, its individuals generally represent similar objective-preference directions. Neighborhood-based parent selection maintains local-search continuity while enabling adjacent subproblems to share useful structural information. Specifically, the current representative of subproblem i and the representatives of its neighboring subproblems form the candidate parent set. Two parent molecules are selected from this set for latent-space crossover and mutation. Their similar preference directions reduce excessively large search perturbations, while their structural variation provides combinatorial diversity for offspring generation.

**Figure 5:**
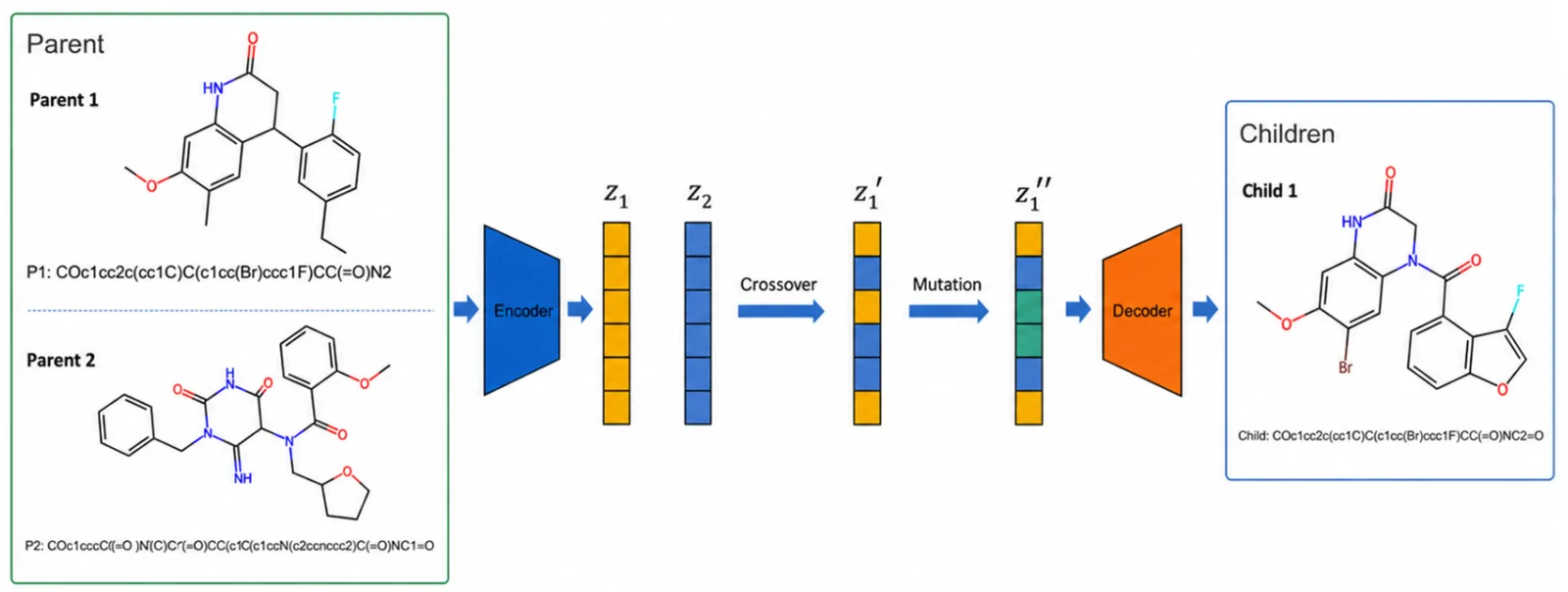
Latent-Space-Based Offspring Generation in MoDeMol. The vectors $z_{1}$ and $z_{2}$ represent the encoded parents, whereas $z_{1}^{\prime }$ and $z_{1}^{\prime \prime }$ denote the offspring representations after crossover and mutation, respectively.

As shown in Figure 5, MoDeMol does not directly perform crossover and mutation on discrete SMILES strings. Instead, it maps two parent molecules into the continuous latent space through the encoder. Let their latent vectors be $z_{1}$ and $\;z_{2}$, respectively. The algorithm first generates a candidate offspring latent vector through linear crossover:

(18) $${z^{\prime }}_{1}=z_{1}+\left(-d+\left(1+2d\right)\alpha \right)\left(z_{2}-z_{1}\right)$$

where α is a random crossover coefficient that controls the position of the offspring latent vector ${z^{\prime }}_{1}$ between the two parents. Parameter d controls interpolation or extrapolation in the blended linear crossover operator and was set to 0.5 in our experiments. This operation combines latent characteristics from both parents while retaining search flexibility. To enlarge the local search region, the algorithm further applies Gaussian mutation to the candidate latent vector:

(19) $${z^{\prime \prime }}_{1}={z^{\prime }}_{1}+\eta$$

where η denotes a random perturbation sampled from a Gaussian distribution. Gaussian mutation is used to enhance local exploration capability and reduce the risk of repeatedly generating highly similar candidate molecules. Compared with discrete string editing, continuous perturbation in the latent space provides a smoother search process. Nevertheless, the resulting latent vector still needs to be decoded and subjected to chemical validity checking.

The mutated latent vector ${z^{\prime \prime }}_{1}$ is converted into a SMILES representation through the decoder. The algorithm then performs chemical validity checking on the decoded result, including whether the molecule can be correctly parsed and whether it satisfies basic chemical structural constraints. Only offspring molecules that pass the validity check are allowed to enter the property evaluation stage and participate in subsequent population updating and elite archive maintenance.

#### 4.4.3. Similarity-Penalized Population Updating

For multi-objective optimization, the retention of candidate molecules is commonly determined by their objective-function performance with respect to the corresponding subproblems. However, in molecular optimization tasks, candidate molecules with comparable property scores may exhibit highly similar molecular scaffolds. If population updating is driven solely by objective fitness, structurally similar molecules may be repeatedly retained within local neighborhoods, leading to a gradual reduction in the structural diversity of the candidate set. To address this limitation, MoDeMol incorporates a molecular-fingerprint-based similarity penalty into the scalarized fitness during the environmental selection stage, thereby forming a composite fitness criterion that jointly evaluates the objective optimization quality and the degree of structural redundancy of candidate molecules.

Within this framework, each subproblem is represented by a weight vector, corresponding to a specific objective-preference direction. For candidate molecule *x*, let f(x) denote its objective vector, $z^{*}$ the current ideal point, and $w_{j}$ the weight vector of the *j*th subproblem. MoDeMol computes the corresponding scalarized fitness component using the following function:

(20) $$g\left(x|w_{j},z^{*}\right)=\left(f\left(x\right)-z^{*}\right)w_{j}$$

where $f\left(x\right)-z^{*}$ denotes the objective-wise difference between the candidate molecule and the current ideal point, and $w_{j}$ is used to weight different objectives according to the preference direction of the corresponding subproblem. This scalarized fitness component transforms the multi-objective property vector into a subproblem-specific scalar value, enabling different candidate molecules to be compared under the same objective- preference direction.

**Molecular fingerprint similarity calculation**

Molecular fingerprints provide a fixed-length feature representation of molecular structures and can be used to efficiently compare structural similarity between different molecules. In this study, Morgan molecular fingerprints are employed to represent the local structural characteristics of candidate molecules. Morgan fingerprints iteratively encode molecular structures based on atom-neighborhood information, thereby capturing local substructures, connectivity patterns, and functional-group motifs within molecules. Therefore, they are suitable for measuring the structural proximity between candidate molecules.

After obtaining the molecular fingerprints, MoDeMol uses the Tanimoto coefficient to calculate the structural similarity between two molecules. For a candidate molecule x, its molecular fingerprint is denoted as FP(x). For the representative solution $x^{k}$ currently associated with the *k* subproblem, its molecular fingerprint is denoted as $FP(x^{k})$. The structural similarity between the two molecules is then expressed as: $\mathrm{Tanimoto}(FP\left(x\right),FP\left(x^{k}\right))$. A higher Tanimoto coefficient indicates stronger structural similarity between the two molecules. To characterize the degree of structural redundancy of a candidate molecule with respect to its local neighborhood, MoDeMol further computes the maximum fingerprint similarity between the candidate molecule and the representative solutions in the neighborhood:

(21) $${sim}_{max}\left(x,B\left(j\right)\right)={max}_{k\in B(j)}\mathrm{Tanimoto}\left(FP\left(x\right),FP\left(x^{k}\right)\right)$$

where B(j) denotes the neighborhood index set of the *j* subproblem, and $x^{k}$ represents the current representative solution associated with the *k* neighboring subproblem. If this value is relatively large, the candidate molecule is structurally close to at least one representative solution in the neighborhood and thus exhibits a high degree of local structural redundancy. Conversely, a smaller value indicates that the candidate molecule provides greater structural variation.

**Similarity-penalized fitness for environmental selection** During the environmental selection stage, MoDeMol combines the scalarized fitness term with the molecular-fingerprint similarity term to form a composite fitness criterion. For a candidate molecule *x* and the subproblem *j* to be updated, the composite fitness is defined as

(22) $$G\left(x|w_{j},z^{*}\right)=g\left(x|w_{j},z^{*}\right)+\lambda_{t}{sim}_{max}\left(x,B\left(j\right)\right)$$

where $\lambda_{t}$ denotes the dynamic similarity-penalty coefficient at generation *t*, which controls the influence of structural similarity during environmental selection. When a candidate molecule is highly similar to existing representative solutions in the neighborhood, the similarity penalty term increases its composite fitness value, thereby placing it at a disadvantage in the replacement decision. Conversely, candidate molecules with favorable objective performance and low neighborhood similarity are more likely to be retained.

The similarity-penalty coefficient is dynamically adjusted during the evolutionary process:

(23) $$\lambda_{t}=\lambda_{max}{\left(\frac{t}{T}\right)}^{\gamma }$$

where $\;\lambda_{max}$ denotes the maximum penalty intensity, *T* denotes the maximum number of iterations, and γ is a growth-control parameter. In the early stage of evolution, $\lambda_{t}$ is relatively small, and the algorithm primarily relies on objective-function performance to advance the search. As the iteration proceeds, $\lambda_{t}$ gradually increases, strengthening the influence of structural similarity in environmental selection and thereby more effectively suppressing the repeated retention of highly similar molecules.

MoDeMol adopts a neighborhood-based updating strategy. For each valid offspring generated by an active subproblem, the algorithm uses it to attempt to update the representative solution of the active subproblem and those of its neighboring subproblems. Specifically, for each subproblem to be updated within the neighborhood, the algorithm computes the composite fitness values of both the candidate offspring and the current representative solution. If the candidate offspring achieves a more favorable composite fitness value, it replaces the current representative solution; otherwise, the original representative solution is retained. In this way, a valid offspring can update one or more neighborhood representative solutions according to the weight vectors of different neighboring subproblems and their local similarity conditions.

#### 4.4.4. Elite Archiving and Adaptive Resource Updating

To preserve high-quality candidate molecules generated during the search process, MoDeMol maintains an external elite archive A. This archive is used to record valid non-dominated molecules, enabling the algorithm to retain historical candidates with favorable overall properties throughout the iterative optimization process. Compared with the current population, the elite archive is not constrained by the position of any single subproblem and can preserve candidate molecules originating from different objective trade-off directions.

After a valid offspring molecule has completed property evaluation, the algorithm compares it with the existing molecules in the elite archive in terms of dominance relationships. If the offspring is not dominated by any molecule in the archive, it is added to the elite archive. If the offspring dominates some existing archive molecules, the dominated archive molecules are removed. Through this update rule, the elite archive continuously maintains the set of non-dominated candidates discovered during the search process.

When the size of the elite archive exceeds the predefined capacity limit $A_{max}$, the algorithm further filters the non-dominated molecules in the archive according to crowding distance. Crowding distance is used to quantify the local distribution density of candidate molecules in the objective space. Molecules with larger crowding distances are located in relatively sparse objective regions and are therefore more valuable for maintaining Pareto-frontier coverage. Consequently, when the archive capacity is limited, MoDeMol preferentially retains non-dominated molecules with larger crowding distances. By combining dominance relationships with crowding distance, the elite archive ensures both the multi-objective superiority of candidate molecules and the distributional diversity of the candidate set in the objective space.

**Statistics of subproblem evolutionary success events**

To track recent replacement success, the algorithm initializes a counter $c_{i}$ to zero for each subproblem *i* at the start of each resource-scheduling period. The counter is incremented whenever an offspring successfully replaces the current representative of that subproblem.

**Calculation of evolutionary activity**

Every $T_{r}$ generations, the algorithm calculates the evolutionary activity of each subproblem from the success counts accumulated during the previous scheduling period and normalizes the activity into a resource-allocation probability:

(24) $$p_{i}=\frac{c_{i}}{\sum_{j=1}^{N}c_{j}}$$

where $T_{r}$ denotes the resource-scheduling period and *N* denotes the number of subproblems. A larger $p_{i}$ indicates that subproblem *i* was more likely to produce effective replacements during the most recent period and therefore has a higher probability of receiving reproduction opportunities in the next stage.

## 5. Conclusions

This study addresses objective conflicts, structural redundancy among candidate molecules, and insufficient search efficiency under limited evaluation budgets in multi-objective lead optimization. To this end, we propose MoDeMol, a decomposition-based evolutionary framework for molecular optimization. MoDeMol constructs a score-informed latent space that encourages molecules with similar predicted target-activity scores to acquire similar latent representations and uses molecular-bank-assisted population initialization to balance exploration around the lead with access to high-quality regions. It then decomposes the multi-objective optimization task into a set of cooperative subproblems defined by weight vectors and performs parent selection, crossover and mutation, environmental selection, and elite archive maintenance in the continuous latent space. By incorporating molecular-fingerprint-based similarity penalization and adaptive resource allocation, MoDeMol improves multi-objective molecular optimization through structural-redundancy control and search-resource scheduling.

Experimental results show that MoDeMol performs favorably against MolFinder, JANUS, and MOMO on both the GSK3β single-target four-objective task and the GSK3β/JNK3 dual-target five-objective task. Latent-space visualization and interpolation analyses indicate that the score-informed latent space produces a more structured distribution of predicted target-activity scores and smoother property transitions along representative interpolation paths. Ablation studies further support the contributions of molecular-fingerprint-based similarity penalization and adaptive resource allocation. Scaffold analysis also shows that MoDeMol generates candidate molecules containing scaffolds that are absent from the corresponding initial populations.

It should be noted that the experiments in this study are still mainly based on predicted properties and fixed-threshold tasks. The actual pharmacological activity, target selectivity, and synthesizability of the obtained candidate molecules still require further experimental validation. Future work may incorporate more accurate property prediction models to further improve the reliability and practical value of multi-objective molecular optimization in drug discovery. 

## Figures and Tables

**Table 1:** Success criteria for the two optimization tasks.

Task	Success Criterion
Task1	QED (>0.7), GSK3β (>0.4), SA_norm (>0.7), Similarity (>0.2)
Task2	QED (>0.7), GSK3β (>0.4), JNK3 (>0.4), SA_norm (>0.7), Similarity (>0.2)

**Table 2:** Performance comparison on Task 1. HV is reported as the mean ± standard deviation across 100 lead molecules.

Model	HV	SR (%)	Diversity	Number
MoDeMol	0.3148±0.1071	96	0.6229	705
MOMO	0.2814±0.1433	82	0.5421	680
MolFinder	0.1970±0.1512	50	0.4591	221
JANUS	0.1551±0.1273	29	0.2884	82

**Table 3:** Performance comparison on Task 2. HV is reported as the mean ± standard deviation across 100 lead molecules.

Model	HV	SR (%)	Diversity	Number
MoDeMol	0.2601±0.0924	88	0.5797	755
MOMO	0.1777±0.1327	56	0.4786	462
MolFinder	0.0824±0.1268	23	0.3254	139
JANUS	0.0687±0.0922	16	0.3967	71

**Table 4:** Ablation and control results on the five-objective task. HV and Diversity are reported as the mean ± standard deviation across 100 lead molecules.

Model	HV	SR (%)	Diversity	Number
MoDeMol	0.2601±0.0924	88	0.5797±0.2419	755
MoDeMol-FSP	0.2387±0.0943	82	0.5216±0.2773	554
MoDeMol-ARA	0.2436±0.0931	74	0.6349±0.1877	480
Original-latent-space	0.2569±0.0927	88	0.6101±0.2221	740
Original-MOEA-D	0.2409±0.0982	80	0.5527±0.2599	666

**Table 5:** Scaffold statistics across lead-molecule optimization tasks.

Metric	Result
Mean number of unique scaffolds in the final archive	101.13
Mean number of new scaffolds in the final archive	77.57
Successful molecules with a new scaffold	255/313

## Data Availability

The original data presented in the study are openly available on GitHub at https://github.com/jellyjun/modemol (accessed on 29 September 2026).
